# P-1070. Safety and Efficacy of Replacement with Contezolid-Containing Regimens in Patients with Multidrug-Resistant/Rifampicin-Resistant Tuberculosis Who are Intolerant to Linezolid-Containing Regimens: a Retrospective Case Analysis

**DOI:** 10.1093/ofid/ofae631.1258

**Published:** 2025-01-29

**Authors:** Tingting Chang, Yuxia Zhang, Yanming Sun, Zhiyuan Zhao, Fengxia Liu, Yu Xiong

**Affiliations:** Department of Tuberculosis VII, Shandong Public Health Clinical Center Affiliated to Shandong University, Jinan, Shandong, China (People's Republic); Department of Tuberculosis VII, Shandong Public Health Clinical Center Affiliated to Shandong University, Jinan, Shandong, China (People's Republic); Department of Tuberculosis VII, Shandong Public Health Clinical Center Affiliated to Shandong University, Jinan, Shandong, China (People's Republic); Department of Tuberculosis VII, Shandong Public Health Clinical Center Affiliated to Shandong University, Jinan, Shandong, China (People's Republic); Department of Tuberculosis VII, Shandong Public Health Clinical Center Affiliated to Shandong University, Jinan, Shandong, China (People's Republic); Department of Tuberculosis VII, Shandong Public Health Clinical Center Affiliated to Shandong University, Jinan, Shandong, China (People's Republic)

## Abstract

**Background:**

Linezolid, commonly used in the treatment of multidrug-resistant tuberculosis (MDR-TB), has limitations due to adverse effects such as myelosuppression, optic neuropathy. Contezolid, a new oxazolidinone, was developed to reduce toxicity. This preliminary study focused on the potential utility of contezolid in the treatment of MDR-TB.

**Figure:**
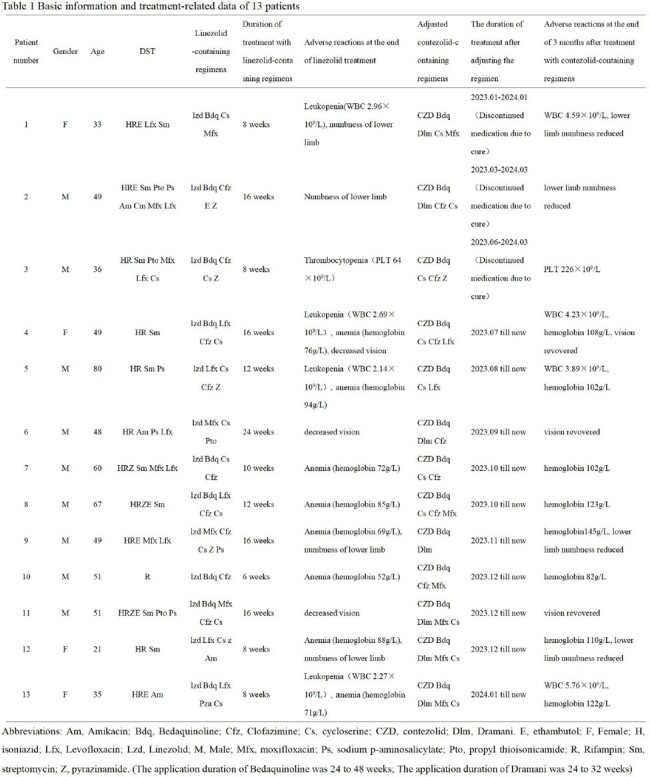

**Methods:**

This is a retrospective case analysis, thirteen MDR/rifampicin-resistant(RR) TB patients who were switched to contezolid-containing regimens due to adverse reactions during treatment with linezolid-containing regimens were included. Linezolid was administered 600mg once daily for 6-24 weeks, contezolid was given 400mg twice daily for 4 months to 1 year. The results of drug sensitivity test (DST), regimens containing linezolid or contezolid and treatment duration, occurrence of adverse reactions, timing of treatment adjustment, adverse reactions and efficacy are described.

**Results:**

The basic information and treatment related results were shown in Table 1. All of 13 patients experienced adverse reactions during the treatment of linezolid-containing regimens, of which 9 (69.2%) were severe. The time to myelosuppression after linezolid initiation was approximately 8 weeks, and 16 weeks for neurotoxicity. After at least 4 weeks of switching to regimens containing contezolid, 95% of patients with linezolid-related adverse reactions were alleviated or improved. At the end of the third month after treatment adjustment, the WBC, PLT and hemoglobin level of 13 patients returned to pre-treatment level, and the lower limb numbness of 2 patients were improved. Sputum mycobacterium tuberculosis culture and sputum smears acid fast stain of 13 patients turned negative or continued negative, and no case of positive recurrence. Follow-up chest computed tomography showed no evidence of disease progression.

**Conclusion:**

This study showed that contezolid were not only safer than linezolid but also demonstrated clinical improvement in all included patients with MDR-TB. This study supports the hypothesis that contezolid may provide an effective and safe alternative to linezolid in the management of MDR-TB in the future.

**Disclosures:**

**All Authors**: No reported disclosures

